# Synthesis and SAR Study of Novel Peptide Aldehydes as Inhibitors of 20S Proteasome

**DOI:** 10.3390/molecules16097551

**Published:** 2011-09-05

**Authors:** Yuheng Ma, Bo Xu, Yuan Fang, Zhenjun Yang, Jingrong Cui, Liangren Zhang, Lihe Zhang

**Affiliations:** State Key Laboratory of Natural and Biomimetic Drugs, School of Pharmaceutical Sciences, Peking University, Beijing 100191, China

**Keywords:** 20S proteasome, inhibitor, peptide aldehydes, synthesis, structure-activity relationship

## Abstract

Based on the analysis of the crystal structure of MG101 (**1**) and 20S proteasomes, a new series of peptide aldehyde derivatives were designed and synthesized. Their ability to inhibit 20S proteasome was assayed. Among them, Cbz-Glu(O*t*Bu)-Phe-Leucinal (**3c**), Cbz-Glu(O*t*Bu)-Leu-Leucinal (**3d**), and Boc-Ser(OBzl)-Leu-Leucinal (**3o**) exhibited the most activity, which represented an order of magnitude enhancement compared with MG132 (**2**). The covalent docking protocol was used to explore the binding mode. The structure-activity relationship of the peptide aldehyde inhibitors is discussed.

## 1. Introduction

Lysosomes and the ubiquitin-proteasome pathway (UPP) are two major routes for cellular protein degradation [1,2,3]. The UPP is essential for many cellular regulatory mechanisms and plays a crucial role in the regulation of many physiological processes. For example, degradation of the p53 tumor suppressors [4] and inhibition of cyclin-dependent p27 kinases [5] can promote tumorigenesis, disorders of protein degradation that originated from UPP can cause the development of many human diseases, such as cancer, Alzheimer’s and Parkinson’s diseases, *etc.* [6,7,8]. Recently, the study of proteasome inhibition has received much attention [9,10,11,12,13,14]. In UPP, proteolysis takes place in the 26S proteasome, which consist of one or two 19S regulatory particles (RP) [15] and a central catalytic particle known as the 20S proteasome (CP). The 20S proteasome is a large cylindrically-shaped complex composed of two copies of seven distinct α- and seven distinct *β*-type subunits [16,17]. It possesses three protease activities, namely the post-glutamyl-peptide hydrolyzing (PGPH), the trypsin-like (T-L), and the chymotrypsin-like (ChT-L) activity, which are assigned as the active subunits *β*1, *β*2, and *β*5, respectively [18,19].

**Figure 1:**
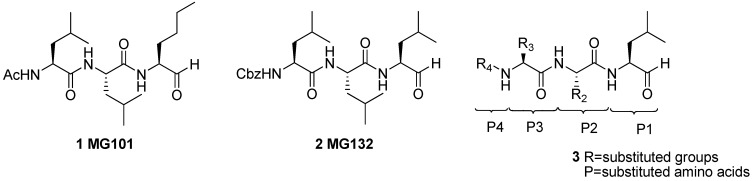
Structures of MG101 (**1**), MG132 (**2**) and peptide aldehydes (**3**).

Small molecules, have been developed to inhibit the proteasome such as cyclic peptides [10,20,21,22], peptide boric acids [23], peptide epoxides [24], peptide vinyl sulfones [25], and nonpeptidic molecules [26,27,28,29]. Among all the proteasome inhibitors ever studied, peptide aldehydes were the first developed and are still the most widely used in *in vitro* and *in vivo* studies [30]. MG101 (**1**, Ac-Leu-Leu-*n*Leu-al, Figure 1), one of calpain inhibitors, is the first well-known 20S proteasome inhibitor [16,17,31]. The crystal structure of the 20S proteasome in complex with MG101 confirms that the hydroxyl group of the N-terminal threonine of the *β*5 subunit reacted with the aldehyde group and formed a reversible hemiacetal. MG132 (**2**, Cbz-Leu-Leu-Leu-Al, Figure 1), a more potent and selective analog of MG101, which bears a benzyloxycarbonyl group instead of an acetyl group, is one of the most commonly used synthetic proteasome inhibitors [32,33]. Up to now, many peptide aldehydes have been designed and synthesized [34,35].

Previous studies have demonstrated that hydrophobic groups around the P1 and P3 positions are beneficial to enhance the activity of peptide aldehydes **3** [36,37,38]. Bulky substituents at the P2 position and aromatic groups at P4 position also contribute to enhance the inhibitory activity [37]. According to the crystal structure of complexed MG101 and 20S proteasome, the leucine side chain of P3 projects into the S3 pocket of *β*5 subunit, which is an open space in the vicinity of the isopropyl groups. Since the P3-leucine moiety only partially fills the S3 pocket, we supposed that introducing a large group at P3 to fill the open space might enhance inhibitory activity. Thus, in this study, we mainly focus on the variation of P3 position to reveal the structure-activity relationships. A series of peptide aldehyde derivatives are designed which have a bulky P3 moiety aiming to increase the hydrophobic interactions with S3.

## 2. Results and Discussion

### 2.1. Synthesis of Peptide Aldehydes ***3a-r***

**Scheme 1:**
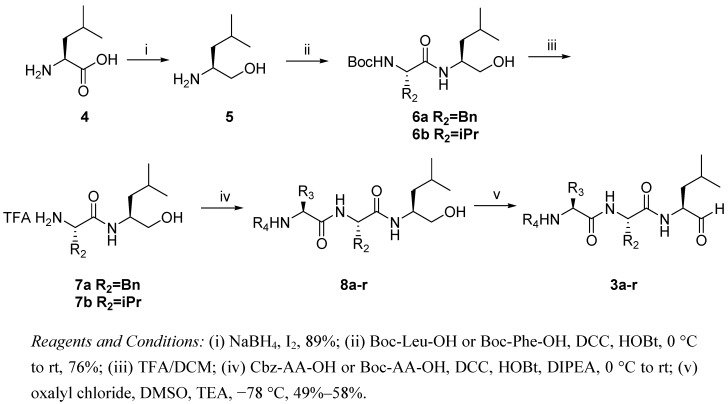
Synthesis of peptide aldehydes **3**.

The synthesis of the peptide aldehydes is shown in Scheme 1. L-Leucine (**4**) was treated with NaBH_4_ and I_2_ under argon to give L-leucinol (**5**) in 89% yield [39], which was then coupled with Boc-protected amino acids to form the dipeptide alcohols **6** in 71%–80% yield. The dipeptide alcohold were deprotected with 20% trifluoroacetic acid in dichloromethane, followed by reaction with *t*-butoxy-carbonyl (Boc)- or benzyloxycarbonyl (Cbz)-protected amino acids to give **8a–r** (crude products were used in the next step without further purification). After Swern oxidation [40], compounds **3a–r** were obtained in 49%–59% yields. 

### 2.2. Assays for Proteasome Activities and SAR

Inhibitory activities of peptide aldehydes on the 20S proteasome are assayed *in vitro* [41]. MG132 was used as the positive control (Table 1). The results indicate that most of the peptide aldehydes exhibited inhibitory activities against ChT-L, which is closely associated with the substituted amino acids at P3. Out of 17 synthesized compounds, nine exhibited inhibitory activities with IC_50_ in the *n*M range, and three compounds in particular (**3c**, **3d**, and **3o**) demonstrated much higher activities than the control MG132.

These inhibitors can be classified into Cbz and Boc series, based on the moiety at the P4 position (R_4_). Among the Cbz series, a P3 residue with a bulky hydrophobic branch (compounds **3a**–**3e**, **3h**) affords a highly active inhibitor, whereas, the electropositive branches (compounds **3f** and **3g**) show dramatically decreased activity. Compounds **3c** and **3d** exhibited about 10-fold higher activity than the others, indicating that a relatively long bulky side chain may favor the increase of activity. For the Boc series, the P3 residue with a hydrophobic bulky side chain generally affords an active ChT-L inhibitor. The activities of Boc series compounds with different P3 residues go in an order of **3o** > **3p** > **3i** and **3k** > **3j** > **3l**, also showing that a bulky side chain is too long to give higher activity. Both **3m** and **3n** show poor activities with IC_50_ > 50 μM, and the reason for this deceased potency might be derived from the presence of a proline pyrrolidine moiety at the P3 position, which is consistent with reported results [37]. Furthermore, in contrast to the Cbz series, in which the activity does not vary obviously with the side chain of P2, in the Boc series, a benzyl group at P2 (**3i** and **3k**) affords much higher activity than the corresponding isobutyl branched compound (**3j **and **3l**). 

**Table 1:** Inhibition of peptide aldehydes to ChT-L activity of 20S proteasome.

Compounds	R4	P3	P2	ChT-L（IC_50,_μM）
**3a**	Cbz	Asp(O*^t^*Bu)	Phe	0.21 ± 0.014
**3b**	Cbz	Asp(O*^t^*Bu)	Leu	0.20 ± 0.035
**3c**	Cbz	Glu(O*^t^*Bu)	Phe	0.028 ± 0.006
**3d**	Cbz	Glu(O*^t^*Bu)	Leu	0.089 ± 0.02
**3e**	Cbz	Phe	Leu	0.85 ± 0.047
**3f**	Cbz	Arg(NO_2_)	Leu	>50
**3g**	Cbz	Arg(Tos)	Leu	>50
**3h**	Cbz	Nap^a^	Leu	0.41 ± 0.082
**3i**	Boc	Asp(OBzl)	Phe	4.83 ± 2.30
**3j**	Boc	Asp(OBzl)	Leu	20.3 ± 2.05
**3k**	Boc	Glu(OBzl)	Phe	7.14 ± 1.93
**3l**	Boc	Glu(OBzl)	Leu	>50
**3m**	Boc	Pro	Phe	>50
**3n**	Boc	Pro	Leu	>50
**3o**	Boc	Ser(OBzl)	Leu	0.050 ± 0.002
**3p**	Boc	Thr(OBzl)	Leu	0.29 ± 0.021
**3q**	Boc	Tyr(OBzl)	Leu	>50
MG132 ( **3r**)	Cbz	Leu	Leu	0.28 ± 0.06

^a^ (2-naphthyl)-L-alanine.

**Figure 2:**
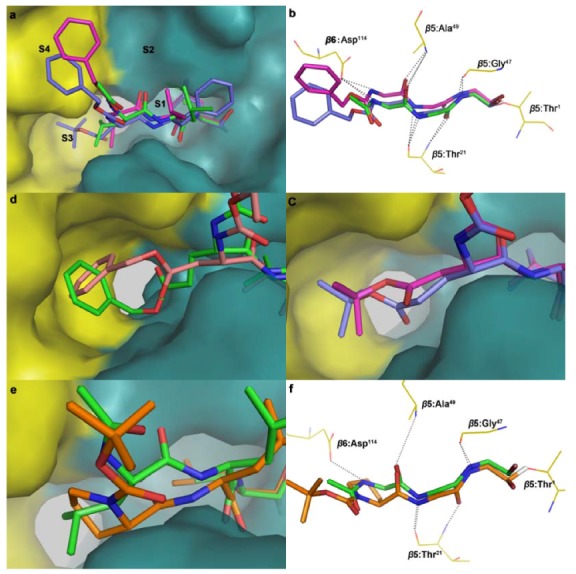
(**a**) Binding modes of **1** (green), **2** (magenta) and **3c** (purple) with 20S proteasome. *β*5 and *β*6 subunits are shown in green and yellow, respectively. (**b**) The binding sites of proteasome and **1** (green), **2** (magenta) and **3c** (purple). Only backbones and key residues of active sites are shown. (**c**) Binding modes of **3a** which is shown in magenta and **3c** in purple. The side chains at P3 position project into S3 pocket. (**d**) Binding modes of **3j** **(**pink) and **3l** (green). The side chains at the P3 position project into S3 pocket. (**e**) Chymotryptic-like active site in binding modes with **1** (green) and **3m** (orange). (**f**) Key contacts between residues of the ligand binding site of the 20S proteasome core particle and the backbones of **1** (green) and **3m** (orange).

To fully understand the SAR of inhibitors, we constructed a binding mode of the peptide aldehydes with the *β*5 subunit of the 20S proteasome based on the crystal structure of 20S proteasome complexed with MG101. Though docking and biochemical data are often not easily comparable, the insights gained into the binding behavior by molecular modeling is meaningful. Given that covalent binding is a unique feature of peptide aldehyde inhibitors, we adopted a covalent docking approach and then developed a protocol to investigate the binding mode of peptide aldehydes with the 20S proteasome. The binding mode of the control MG132 is similar to that of MG101 observed in the crystal structure (Figure 2a). MG132 adopts a *β*-conformation and fills the gap between strands S2 and S4 by forming hydrogen bonds with residues Thr21, Gly47, and Ala49 and generating an anti-parallel *β*-sheet structure (Figure 2b) [10]. The P1-leucine side chain of MG132 projects into the S1 pocket and the P2-leucine side chain is towards outside. The P3-leucine side chain stretches out into the subunit-specific S3 pocket and is in close contact with residues of the adjacent *β*6 subunit. 

Other peptide aldehydes are also docked into the 20S proteasome using the same protocol. Similar orientations of P1–P4 residues are found in the docked conformations. For example, the P1–P4 residues of **3c** are towards the S1–S4 pockets, respectively, like in MG132 (Figure 2a), and so do the hydrogen bonds (Figure 2b).

Biochemical investigation shows that the size and length of the P3 side chain is crucial to activity [42,43]. Among the Cbz series compounds, Glu(O^t^Bu) residues at P3 (**3c** and **3d**) give the most active inhibition. The structure of the 20S proteasome shows that the *β*5 and *β*6 subunits constitute the binding cleft of the S3 pocket, which is able to accommodate long and linear side chains. The docking results show that the *tert*-butyl glutamic ester (**3c**) fits this site better than *tert*-butyl aspartic ester (**3a**) and provides a strong hydrophobic interaction with the *β*5/*β*6 interface (Figure 2c). Among the Boc series of compounds, when a phenyl ester was used to replace a *tert*-butyl ester at P3, the Asp(OBzl) residue (**3j**) exhibited more active inhibition than Glu(OBzl) (**3l**). According to the docking analysis (Figure 2d), although the phenyl ester of both Asp(OBzl) and Glu(OBzl) project into the S3 pocket, the large sized benzene ring makes the conformation rigid and pushes the backbone slightly out of the original orientation, so the shorter side chain of Asp(OBzl) is more suitable for the cleft. The most suitable length of side chain in this Boc-series is Ser(OBzl), and it gives most active inhibition to ChT-L. When the residue at the P3 position is changed to proline (**3m** and **3n**), the results show that the pyrrolidine moiety projects into the S3 pocket (Figure 2e), which makes the binding model of the main chain different from that of MG132 (Figure 2f), and resulting in the disappearance of activity.

## 3. Experimental

### 3.1. Chemistry

#### 3.1.1. General

Unless specified otherwise, all starting materials and reagents were used as obtained from commercial suppliers without further purification. Thin layer chromatography was performed using silica gel GF-254 (Qing-Dao Chemical Company, China) plated with detection by UV, and column chromatography was performed on silica gel (200–300 mesh, Qing-Dao Chemical Company, China). Optical rotations were recorded on a Perkin-Elmer 243B polarimeter. ^1^H-NMR (300 or 500 MHz) spectra was recorded on Varian VXR-300 and Varian Inova VXR-500 spectrometer. Mass spectra (ESI-TOF^+^ MS) was obtained on a MDS SCLEX QSTAR instrument and only the most representative peaks were reported (*m/z*).

#### 3.1.2. Synthesis

*L-Leucinol* (**5**) [39]. Sodium borohydride (1.42 g, 37 mmol, 2.4 eq.) was dissolved in anhydrous THF (40 mL) and L-leucine (2.00 g, 15 mmol, 1 eq.) was added in one portion. The solution was cooled to −5 °C in an ice-salt bath, and a solution of iodine (3.87 g, 15 mmol, 1 eq.) in anhydrous THF (10 mL) was added dropwise over 40 min. After the gas evolution was ceased, the reaction solution was refluxed for 16 h. The solution was cooled to room temperature and methanol was added cautiously until the mixture became clear. After stirring 30 min, the solution was evaporated and the residue was dissolved by addition of 45 mL aqueous NaOH. The solution was stirred for 2.5 h and extracted with methylene chloride (30 mL × 4). The combined organic extracts were dried over Na_2_SO_4_ and concentrated, affording crude product which was distilled under reduced pressure to yield colorless oil **5** (1.59 g, 89.3%, 80–83 °C/25 mmHg). 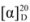 +4.0 (c 0.3, MeOH). ^1^H-NMR (500 MHz, CDCl_3_): δ 0.91 (d, 6H, *J* = 6.5 Hz), 1.19–1.14 (m, 2H), 1.70–1.65 (m, 1H), 2.04 (*br* s, 3H, NH_2_, OH), 3.23 (dd, 1H, *J* = 3.0, 8.0 Hz), 3.57 (dd, 1H, *J* = 3.0, 8.0 Hz). MS (ESI-TOF^+^): 118 [M+H]^+^.

*Boc-L-Phenylalanine-L-Leucinol* (**6a**). Boc-L-phenylalanine (0.25 g, 0.95 mmol, 1.0 eq.), L-leucinol (0.20 g, 1.04 mmol, 1.0 eq.), and HOBt (0.14 g, 1.0 mmol, 1.1 eq.) were mixed in anhydrous THF (2 mL). N,N`-Dicyclohexylcarbodiimide (DCC, 0.22 g, 1.0 mmol, 1.1 eq.) and **5** (0.11 g, 0.95 mmol, 1 eq.) was added at 0 °C, and the mixture was warmed to room temperature and stirred for 16 h. After filtration to remove dicyclohexylurea, the solvent was removed and the residue was partitioned between EtOAc (20 mL) and H_2_O (10 mL). The organic phase was washed with 10% citric acid (10 mL × 3), saturated NaHCO_3_ (10 mL × 3), and then brine (10 mL × 2). The solution was dried over Na_2_SO_4_ and evaporated to an amorphous solid. The crude product was purified by flash chromatography on silica gel to give compound **6a** as white solid (0.25 g, 71.2%). 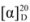 −19.4 (c 0.3, MeOH). ^1^H-NMR (500 MHz, CDCl_3_): δ 0.87 (d, 6H, *J* = 7.2 Hz), 1.26–1.23 (m, 2H), 1.42 (s, 9H), 1.50–1.46 (m, 1H), 1.95 (s, 1H), 3.02–2.98 (m, 1H), 3.11–3.07 (m, 1H), 3.35 (m, 1H), 3.48 (d, 1H, *J* = 11.0 Hz), 3.95–3.93 (m, 1H), 4.25 (m, 1H), 5.06 (s, 1H), 5.60 (d, 1H), 7.28–7.14 (m, 5H). MS(ESI-TOF^+^): 345 [M+H]^+^.

*Boc-L-Leucine-L-Leucinol* (**6b**). Compound **6b** was obtained by using similar procedure as **6a**. White solid. 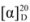 −11.6 (c 0.52, MeOH). ^1^H-NMR (500 MHz, CDCl_3_): δ 0.93–0.88 (m, 12H), 1.32–1.27 (m, 1H), 1.40–1.36 (m, 1H), 1.42 (s, 9H), 1.64–1.60 (m, 4H), 2.63 (s, 1H), 3.51–3.46 (m, 1H), 3.69–3.63 (m, 1H), 4.01–3.98 (m, 2H), 4.84 (s, 1H), 6.20 (d, 1H, *J* = 8.0 Hz). MS (ESI-TOF^+^): 331 [M+H]^+^.

*TFA L-Phenylalanine-L-Leucinol* (**7a**). To a suspension of **6a** (0.50 g, 1.27 mmol) in CH_2_Cl_2_ (3 mL) was added TFA (1 mL) at 0 °C. After stirred at room temperature for 2 h, the solution was evaporated and the crude product was used in the next step without purification. *TFA L-Leucine-L-Leucinol* (**7b**) was prepared by a similar procedure.

*Cbz-L-Asp(OtBu)-L-Phe-L-Leuninol* (**8a**). Cbz-L-Asp(OtBu)-OH (0.20 g, 0.62 mmol, 1 eq.) was dissolved in anhydrous THF (5 mL), HOBt (92 mg, 0.68 mmol, 1.1 eq.) and DCC (0.14 g, 0.68 mmol, 1.1 eq.) were added after cooling to 0 °C. The mixture was stirred for 60 min then **7a** (0.21 g, 0.62 mmol, 1 eq.) and diisopropylethylamine (0.22 mL, 1.24 mmol, 2 eq.) were added. The reaction mixture was stirred at room temperature overnight. The insoluble material was filtered off and the solution was washed successively with 10% citric acid, saturated NaHCO_3_ and brine. After drying with Na_2_SO_4_ the solvents were removed under reduced pressure. The crude products were used in the next step without further purification. Compounds **8b-r** were prepared using similar procedures.

*Cbz-L-Asp(OtBu)-L-Phe-L-Leucinal* (**3a**) A solution of oxalyl chloride (63 mg, 0.49 mmol, 1.4 eq.) in CH_2_Cl_2_ (0.4 mL) was cooled to −78 °C, DMSO (45 μL, 0.63 mmol, 1.8 eq.) in CH_2_Cl_2_ (0.1 mL) was added dropwise over 10 min. The resulting mixture was stirred for 30 min at −78 °C. A solution of **8a** (0.20 g, 0.35 mmol, 1 eq.) in CH_2_Cl_2_ (1.5 mL) was then added dropwise over 30 min affording a cloudy mixture which was allowed to stir at −78 °C for 2 h. Triethylamine (0.19 mL, 1.41 mmol, 4 eq.) was then slowly added over 30 min, and the solution was gradually warmed to room temperature. The reaction mixture was washed with saturated aq NaHCO_3_, and brine. The organic phase was dried, filtered and concentrated. The crude product was purified by flash chromatography on silica gel to give compound **3a** as white solid (0.10 g, 52.3%). White solid. 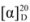 −19.1 (c 0.4, CHCl_3_). ^1^H-NMR (300 MHz, CDCl_3_): δ 0.86 (d, 6H, *J* = 6.2 Hz), 1.40 (s, 9H), 1.70–1.53 (m, 3H), 2.73–2.68 (m, 2H), 3.10–3.04 (m, 2H), 4.38–4.30 (m, 2H), 4.71 (d, 1H, *J* = 7.2 Hz), 5.09 (d, 2H, *J* = 3.6 Hz), 5.72 (m, 1H), 6.58 (d, 1H, *J* = 7.5 Hz), 6.82 (t, 1H, *J* = 8.7 Hz), 7.38–7.19 (m, 10H), 9.43 (d, 1H). MS (ESI-TOF^+^): 568 [M+H]^+^. Elemental Anal.Calcd. for C_31_H_41_N_3_O_7_: C, 65.59; H, 7.28; N, 7.40. found: C, 65.67; H, 7.48; N, 7.32.

Compounds **3b-3r** were obtained using a similar procedure as **3a**.

*Cbz-L-Asp(OtBu)-L-Leu-L-Leucinal* (**3b**): White solid. 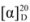 −21.0 (c 0.5, CHCl_3_). ^1^H-NMR (300 MHz, CDCl_3_): δ 0.97–0.88 (m, 12H), 1.42 (s, 9H), 1.69–1.57 (m, 6H), 4.48–4.43 (m, 3H), 5.13 (d, 2H, *J* = 3.6 Hz), 5.88 (s, 1H), 6.67–6.65 (m, 2H), 7.37 (m, 5H), 9.52 (s, 1H). MS (ESI-TOF^+^): 534 [M+H]^+^. Elemental Anal.Calcd. for C_28_H_43_N_3_O_7_: C, 63.02; H, 8.12; N, 7.87. found: C, 63.09; H, 8.19; N, 7.88.

*Cbz-L-Glu(OtBu)-L-Phe-L-Leucinal* (**3c**): White solid. 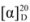 −28.1 (c 0.6, CHCl3). ^1^H-NMR (300 MHz, CDCl_3_): δ 0.90 (d, 6H, *J* = 5.4 Hz), 1.44 (s, 9H), 1.56–1.49 (m, 3H), 1.91–1.88 (m, 2H), 2.25 (t, 2H, *J* = 6.3 Hz), 3.14 (t, 2H, *J* = 8.4 Hz), 4.06–3.96 (m, 1H), 4.38–4.33 (m, 1H), 4.75–4.69 (m, 1H), 5.04 (dd, 2H, *J* = 8.3, 22.2 Hz), 6.02 (s, 1H), 6.70–6.64 (m, 1H), 7.35–7.18 (m, 10H), 9.43 (d, 1H, *J* = 4.8 Hz). MS (ESI-TOF^+^): 582 [M+H]^+^. Elemental Anal.Calcd. for C_32_H_43_N_3_O_7_: C, 66.07; H, 7.45; N, 7.22. found: C, 65.99; H, 7.45; N, 7.26.

*Cbz-L-Glu(OtBu)-L-leu-L-Leucinal* (**3d**): White solid. 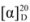 −14.4 (c 0.7, CHCl_3_). ^1^H-NMR (300 MHz, CDCl_3_): δ 0.97–0.90 (m, 6H), 1.44 (s, 9H), 1.74–1.54 (m, 6H), 2.09–1.96 (m, 2H), 2.40 (t, 2H, *J* = 5.1 Hz), 4.18 (t, 1H, *J* = 7.2 Hz), 4.49–4.38 (m, 2H), 5.10 (d, 2H, *J* = 3.3 Hz), 6.14 (s, 1H), 6.62 (s, 1H), 6.96 (s, 1H), 7.35–7.26 (m, 5H), 9.52 (d, 1H, *J* = 9.3 Hz). MS (ESI-TOF^+^): 548 [M+H]^+^. Elemental Anal.Calcd. for C_29_H_45_N_3_O_7_: C, 63.60; H, 8.28; N, 7.67. found: C, 62.54; H, 8.19; N, 7.70.

*Cbz-L-Phe-L-leu-L-Leucinal* (**3e**): White solid, 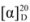 −16.1 (c 0.3, CHCl_3_). ^1^H-NMR (500 MHz, CDCl_3_): δ 0.92 (m, 12H), 1.74–1.32 (m, 6H), 4.36–4.33 (m, 1H), 4.54–4.47 (m, 2H ), 5.04–5.00 (d, 2H, *J* = 12.5 Hz), 5.45–5.40 (m, 1H), 7.00–6.53 (m, 2H), 7.36–7.16 (m, 10H), 9.50 (s, 1H). MS (ESI-TOF^+^): 544 [M+H]^+^. Elemental Anal.Calcd. for C_32_H_37_N_3_O_5_: C, 70.70; H, 6.86; N, 7.73. Found: C, 70.74; H, 6.90; N, 7.65.

*Cbz-L-Arg(NO_2_)-L-leu-L-Leucinal* (**3f**): White solid, 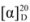 −67.2 (c 0.1, CHCl_3_). ^1^H-NMR (500 MHz, CDCl_3_): δ 0.88–0.86 (m, 12H), 1.66–1.38 (m, 8H), 1.80–1.75 (m, 2H), 3.28–3.24 (m, 2H), 4.07 (dd, 1H, *J* = 4.5, 15.5 Hz), 4.20–4.16( m, 1H), 4.39–4.30 (m, 2H), 5.08 (s, 2H), 5.98 (s, 1H), 6.49–6.33 (m, 1H), 7.35–7.30 (m, 6H), 7.47 (s, 1H), 8.45 (s, 1H), 9.52 (s, 1H). MS (ESI-TOF^+^): 564 [M+H]^+^. Elemental Anal.Calcd. for C_26_H_41_N_7_O_7_, C, 55.40; H, 7.33; N,17.40. Found: C, 55.32; H, 7.42; N, 17.30.

*Cbz-L-Arg(Tos)-L-leu-L-Leucinal* (**3g**): White solid. 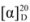 −12.3 (c 0.2, CHCl_3_). ^1^H-NMR (500 MHz, CDCl_3_): δ 1.11–0.86 (m, 12H), 1.74–1.31 (m, 8H), 1.95–1.92 (m, 2H), 2.42 (s, 3H), 2.49–2.47 (m, 2H), 4.20–4.15 (m, 1H), 4.32–4.26 (m, 2H), 5.15 (s, 2H), 5.98 (s, 1H), 6.49–6.43 (m, 1H), 7.38–7.25 (m, 6H), 7.81 (d, 1H), 8.11 (s, 1H), 9.47 (s, 1H). MS (ESI-TOF^+^): 673 [M+H]^+^. Elemental Anal.Calcd. for C_33_H_48_N_6_O_7_S, C, 58.91; H, 7.19; N, 12.49. found: C, 58.99; H, 7.12; N,12.36.

*Cbz-L-(2-Naphthyl)alanine-L-Leu-L-Leucinal* (**3h**): White solid. 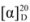 −51.1 (c 0.7, CHCl_3_). ^1^H-NMR (500 MHz, CDCl_3_): δ 0.93–0.89 (m, 12H), 1.91–1.46 (m, 6H), 3.22–3.16 (dd, 2H, *J* = 3.9, 7.5 Hz), 4.48–4.43 (m, H), 4.89–4.75 (m, 2H), 5.12 (d, 2H, *J* = 4.2 Hz), 6.03 (d, 1H), 6.69–6.58 (m, 2H), 7.38–7.31 (m, 6H), 7.51 (t, 1H, *J* = 7.5 Hz), 7.58–7.55 (m, 1H), 7.77 (d, 1H, *J* = 8 Hz), 7.88 (d, 1H, *J* = 8.0 Hz), 8.26 (d, 1H, *J* = 9.0 Hz), 9.51 (d, 1H, *J* = 6.0 Hz). MS (ESI-TOF^+^): 560 [M+H]^+^. Elemental Anal.Calcd. for C_33_H_41_N_3_O_5_: C, 70.82; H, 7.38; N, 7.51. found: C, 70.90; H, 7.42; N, 7.61.

*Boc-L-Asp(OBzl)-L-Phe-L-Leucinal* (**3i**): White solid. 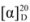 −19.5 (c 0.65, CHCl_3_). ^1^H-NMR (300 MHz, CDCl_3_): δ 0.94–0.88 (m, 6H), 1.40 (s, 9H), 1.60–1.54 (m, 1H), 1.72–1.66 (m, 2H), 3.04–2.77 (m, 3H), 3.26–3.23 (m, 1H), 4.18–4.10 (m, 2H), 4.71–4.67 (m, 1H), 5.11–5.04 (m, 2H), 5.44 (m, 1H), 6.59 (s, 1H), 6.80 (s, 1H), 7.35–7.21 (m, 10H), 9.40 (s, 1H). MS (ESI-TOF^+^): 568 [M+H]^+^. Elemental Anal.Calcd. for C_31_H_41_N_3_O_7_: C, 65.59; H, 7.28; N, 7.40. Found: C, 65.38; H, 7.19; N, 7.47.

*Boc-L-Asp(OBzl)-L-Leu-L-Leucinal* (**3j**): White solid. 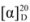 −44.5 (c 0.7, CHCl_3_). ^1^H-NMR (300 MHz, CDCl_3_): δ 0.95–0.92 (m, 12H), 1.44 (s, 9H), 1.70–1.59 (m, 6H), 2.92 (t, 2H, *J* = 5.7 Hz), 4.47–4.40 (m, 3H), 5.22 (s, 2H), 5.48 (d, 1H), 6.70 (d, 1H, *J* = 6.9 Hz), 6.87 (m, 1H), 7.40–7.36 (m, 5H), 9.50 (s, 1H). MS (ESI-TOF^+^): 534 [M+H]^+^. Elemental Anal.Calcd. for C_28_H_43_N_3_O_7_: C, 63.02; H, 8.12; N, 7.87. found: C, 62.65;H, 7.98;N, 7.95.

*Boc-L-Glu(OBzl)-L-Phe-L-Leucinal* (**3k**): White solid. 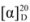 −18.1 (c 0.7, CHCl_3_). ^1^H-NMR (300 MHz, CDCl_3_): δ 0.90–0.87 (m, 6H), 1.37 (s, 9H), 1.69–1.54 (m, 3H), 2.07–2.03 (m, 2H), 2.44 (t, 2H, *J* = 4.2 Hz), 3.21 (dd, 2H, *J* = 7.2, 14.1 Hz), 4.05–4.01 (m, 1H), 4.38–4.29 (m, 1H), 4.77–4.74 (m, 1H), 5.13 (d, 2H, *J* = 3.3 Hz), 5.57 (s, 1H), 6.68–6.56 (m, 2H), 7.38–7.20 (m, 10H), 9.41 (d, 1H, *J* = 5.4 Hz). MS (ESI-TOF^+^); 582 [M+H]^+^. Elemental Anal.Calcd. for C_32_H_43_N_3_O_7_: C, 66.07; H, 7.45; N, 7.22. found: C, 65.91; H, 7.56; N, 7.29.

*Boc-L-Glu(OBzl)-L-Leu-L-Leucinal* (**3l**): White solid. 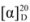 −31.5 (c 0.8, CHCl_3_). ^1^H-NMR (300 MHz, CDCl_3_):δ 0.95–0.90 (m, 12H), 1.43 (s, 9H), 1.97–1.62 (m, 6H), 2.17–2.12 (m, 2H), 2.57–2.48 (m, 2H), 4.11 (t, 1H, *J* = 5.7 Hz), 4.48–4.43 (m, 2H), 5.13 (s, 2H), 5.49 (d, 1H), 6.83 (s, 1H), 7.37–7.27 (m, 5H), 9.52 (s, 1H). MS (ESI-TOF^+^): 548 [M+H]^+^. Elemental Anal.Calcd. for C_29_H_45_N_3_O_7_: C, 63.60; H, 8.28; N, 7.67. found: C, 63.71; H, 8.19; N, 7.68.

*Boc-L-Pro-L-Phe-L-Leucinal* (**3m**): White solid. 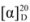 −10.0 (c 0.5, CHCl_3_). ^1^H-NMR (300 MHz, CDCl_3_): δ 0.98–0.88 (m, 6H), 1.43 (s, 9H), 2.08–1.46 (m, 7H), 3.17–3.13 (m, 2H), 3.40–3.35 (m, 2H), 4.45–4.38 (m, 2H), 5.00–4.94 (m, 1H), 5.92 (s, 1H), 6.72 (s, 1H,), 7.40–7.20 (m, 5H), 9.45 (s, 1H). MS (ESI-TOF^+^): 460 [M+H]^+^. Elemental Anal.Calcd. for C_25_H_37_N_3_O_5_: C, 65.34; H, 8.11; N, 9.14. found: C, 65.44; H, 8.19; N, 9.15.

*Boc-L-Pro-L-Leu-L-Leucinal* (**3n**): White solid. 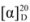 −23.3 (c 0.6, CHCl_3_). ^1^H-NMR (300 MHz, CDCl_3_): δ 1.01–0.93 (m, 12H), 1.50–1.32 (m, 14H), 1.75–1.68 (m, 6H), 4.16–4.12 (m, 1H), 4.52–4.45 (m, 1H), 4.91 (m, 1H), 6.70 (s, 1H), 9.56 (d, 1H, *J* = 2.7 Hz). MS (ESI-TOF^+^): 426 [M+H]^+^. Elemental Anal.Calcd. for C_22_H_39_N_3_O_5_: C, 62.09; H, 9.24; N, 9.87. found: C, 61.18; H, 9.28; N, 9.80.

*Boc-L-Ser(OBn)-L-Leu-L-Leucinal* (**3o**): White solid. 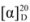 −35.1 (c 0.4, CHCl_3_). ^1^H-NMR (500 MHz, CDCl_3_): δ 0.92–0.88 (m, 12H), 1.80–1.40 (m, 15H), 3.65–3.59 (m, 1H), 3.91–3.86 (m, 1H), 4.52–4.20 (m, 3H), 4.54 (s, 2H), 5.39 (s, 1H), 6.94–6.52 (m, 2H), 7.38–7.25 (m, 5H), 9.52 (*brs*, 1H). MS (ESI-TOF^+^): 506 [M+H]^+^. Elemental Anal.Calcd. for C_27_H_4__3_N_3_O_6_: C, 64.13; H, 8.57; N, 8.31. found: C, 64.10; H, 8.51; N, 8.40.

*Boc-L-Thr(OBn)-L-Leu-L-Leucinal* (**3p**): White solid. 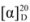 −6.0 (c 0.6, CHCl_3_). ^1^H-NMR (500 MHz, CDCl_3_): δ 0.87–0.84 (m, 6H), 0.99–0.91 (m, 6H), 1.22–1.16 (m, 3H), 1.45 (s, 9H), 1.77–1.50 (m, 6H), 4.19–4.16 (m, 1H), 4.24–4.20 (m, 1H), 4.36–4.27 (m, 1H), 4.51–4.48 (m, 1H), 4.65 (dd, 1H, *J* = 4, 12.0 Hz), 5.45 (s, 1H), 6.87–6.54 (m, 2H), 7.35–7.25 (m, 5H), 9.49 (d, 1H, *J* = 25.0 Hz). MS (ESI-TOF^+^): 520 [M+H]^+^. Elemental Anal.Calcd. for C_28_H_45_N_3_O_6_: C, 64.71; H, 8.73; N, 8.09. found: C, 64.78; H, 8.80; N, 8.01.

*Boc-L-Tyr(OBn)-L-Leu-L-Leucinal* (**3q**): White solid. 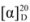 −4.6 (c 0.3, CHCl_3_). ^1^H-NMR (500 MHz, CDCl_3_): δ 0.93 (m,12), 1.40 (s, 9H), 1.76–1.45 (m, 6H), 3.03–3.00 (m, 2H), 4.29–4.22 (m, 1H), 4.46–4.40 (m, 1H), 4.89 (d, 1H, *J* = 4.0 Hz), 5.04 (s, 2H), 6.28 (d, 1H, *J* = 8.5 Hz, NH), 6.36 (m, 1H), 6.74 (s, 1H), 6.92 (dd, 2H, *J* = 6.0, 11.5 Hz), 7.11 (d, 2H, *J* = 8 Hz), 7.43–7.26 (m, 5H), 9.51 (s, 1H). MS (ESI-TOF^+^): 582 [M+H]^+^. Elemental Anal.Calcd. for C_3__3_H_4__7_N_3_O_6_: C, 68.13; H, 8.14; N, 7.22. found: C, 68.07; H, 8.19; N, 7.16. 

*Cbz-L-Leu-L-Leu-L-Leucinal* (MG132, **3r**): White solid. 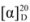 −14.4 (c 0.8, CHCl_3_). ^1^H-NMR (300 MHz, CDCl_3_): δ 0.93–0.88 (m, 18H), 1.65–1.58 (m, 9H), 4.18–4.14 (t, 1H, *J* = 5.5 Hz), 4.47–4.40 (m, 2H), 5.09–4.89 (m, 2H), 5.44 (d, 1H, *J* = 22.5 Hz), 6.66 (d, 1H, *J* = 8.0 Hz), 6.95 (d, 1H, *J* = 33.5 Hz), 7.28–7.38 (m, 5H), 9.52 (d, 1H). MS (ESI-TOF^+^): 476 [M+H]^+^. Elemental Anal.Calcd. for C_26_H_41_N_3_O_5_: C, 65.66; H, 8.69; N, 8.83. found: C, 65.70; H, 8.62; N, 8.79.

### 3.2. Biological Testing

*Assays for proteasome activities.* The enzymatic activities of the proteasome were assayed using fluorogenic peptides: Suc-Leu-Leu-Val-Tyr-AMC (Suc represents succinyl and AMC represents 7-amido-4-methylisocoumarin, obtained from Sigma) for ChT-L activity. 20S proteasome purified from mouse liver (1 μg) was incubated with various concentrations of compounds and 50 μM fluorogenic peptides in 20 mM Tris-HCl pH 7.8 (100 μL) at 37 °C for 1 h, respectively. The fluorescence of released AMC was measured by a spectrofluorimeter (Fluostar OPTIMA, BMG Germany) at excitation/emission wavelengths of 380/440 nm and 335/410 nm, respectively. 0.1% DMSO was used as solvent control. Compared with the fluorescence of solvent control, an inhibition rate was calculated and thereafter the IC_50_ value was deduced.

### 3.3. Molecular Docking

The covalent docking method with Gold 4.0: A radius of 20 Å from the β5-catalytic N-terminal threonine was used to direct site location. For each of the genetic algorithm runs, a maximum number of 100,000 operations were performed on a population of 100 individuals with a selection pressure of 1.1. Operator weights for crossover, mutation, and migration were set to 95, 95, and 10, respectively, as recommended by the authors of the software. 50 GA runs were performed in each docking experiment as done in the software validation procedure. The default GOLD fitness function was used to identify the better binding mode. The distance for hydrogen bonding was set to 2.5 Å and the cut-off value for van der Waals calculation was set to 4 Å. Covalent docking was applied and the terminal carbonyl carbon of all the ligands have been bonded to the hydroxyl oxygen of Thr1.

## 4. Conclusions

Based on the binding analysis of proteasome and its inhibitor, a new series of peptide aldehydes was designed and synthesized. Their abilities to inhibit the 20S proteasome were assayed and the results show that some compounds have more potency than the positive control MG132. Covalent docking was used to simulate the binding of the peptide aldehyde compounds with 20S, and the docking mode is similar to that of the observed crystal complex and that the P3-postion substitutes are crucial for inhibitor potency. The suggested binding mode provides a potential way to design more potent inhibitors of the 20S proteasome.
